# Microdialysis combined with liquid chromatography-tandem mass spectrometry for the quantitation of gemifloxacin and its application to a muscle penetration study in healthy and MRSA-infected rats

**DOI:** 10.1371/journal.pone.0217573

**Published:** 2019-06-06

**Authors:** Rui Zhao, Qing Wang, Xin-Xin Hu, Tong-Ying Nie, Xin-Yi Yang, Cong-Ran Li, Xi Lu, Xiukun Wang, Jian-Dong Jiang, Jing Pang, Xue-Fu You

**Affiliations:** Beijing Key Laboratory of Antimicrobial Agents and Department of Pharmacology, Institute of Medicinal Biotechnology, Chinese Academy of Medical Sciences and Peking Union Medical College, Beijing, China; Macau University of Science and Technology, MACAO

## Abstract

Pharmacological efficacy is based on the drug concentration in target tissues, which usually cannot be represented by the plasma concentration. The purpose of this study was to compare the pharmacokinetic characteristics of gemifloxacin in plasma and skeletal muscle and evaluate its tissue penetration in both healthy and MRSA (methicillin-resistant *Staphylococcus aureus*)-infected rats. A microdialysis (MD) combined with liquid chromatography-tandem mass spectrometry (LC-MS/MS) method was developed and validated to determine free gemifloxacin concentrations in rat plasma and skeletal muscle simultaneously. The *in vivo* recoveries of MD were 23.21% ± 3.42% for skeletal muscle and 20.62% ± 3.19% for plasma, and were concentration independent. We provided evidence that the method developed here meets FDA requirements. Additionally, this method was successfully applied to the determination of free gemifloxacin in rats. Muscle and blood dialysates were collected after an 18 mg/kg intravenous bolus dose. The mean areas under the concentration-time curves (AUCs) from 0 to 9 h for skeletal muscle and plasma were 3641.50 ± 915.65 h*ng/mL and 7068.32 ± 1964.19 h*ng/mL in MRSA-infected rats and 3774.72 ± 700.36 h*ng/mL and 6927.49 ± 1714.86 h*ng/mL in healthy rats, respectively. There was no significant difference (P>0.05) in gemifloxacin exposure between healthy rats and MRSA-infected rats for plasma or muscle. The low ratio of AUC_0-9_ muscle to AUC_0-9_ plasma suggested lower drug exposure in skeletal muscle than in plasma for both healthy and MRSA-infected rats. Our study suggested that the administration of gemifloxacin according to drug levels in plasma to treat local infection is unreasonable and might result in an inadequate dose regimen.

## Introduction

Antibacterial drug resistance, an urgent global crisis, is seriously threatening public health and social stability. The prevalence of methicillin-resistant *Staphylococcus aureus* (MRSA), a major cause of hospital and community-associated infections, has become the focus of novel antibacterial development. Gemifloxacin (pKa_1_ = 5.53, pKa_2_ = 9.53; LogP = 1.7313; molecular weight 389.3809) [1] is a broad-spectrum fluoroquinolone antibacterial with relatively high antimicrobial activity against Gram-positive bacteria. Compared with other quinolones, gemifloxacin shows a better inhibitory effect on topoisomerase IV, a primary target of *Staphylococcus aureus* [2].

The alarming increase in drug resistance is mainly caused by the inappropriate use of antibacterial agents. Pharmacokinetic/pharmacodynamic (PK/PD) studies are a valuable tool to maximize bacteriological cures and minimize drug-resistance mutations in drug development and clinical practice. Quinolones are generally concentration dependent and can rapidly penetrate infected tissue [3–4]. The PK/PD properties of quinolones have been well described, and the ratio of the area under the concentration-time curve to the minimum inhibitory concentration (AUC_0-24_/MIC) has an excellent correlation with clinical efficacy [5]. Pharmacological efficacy is usually related to the free drug concentration in target tissues, but the plasma concentration obtained in pharmacokinetic (PK) studies does not represent the real concentration in the tissue, even taking the protein binding rate into consideration [6]. Therefore, the clinical dosing regimen established based on plasma concentration is questionable. Microdialysis (MD), a technique used to sample protein-free molecules located in a target site by a dialysis membrane, provides the possibility to achieve an appropriate drug concentration in target tissues and is widely utilized in the tissue distribution studies [7–8].

Although gemifloxacin was approved by the U.S. Food and Drug Administration (FDA) only for the treatment of acute bacterial exacerbation of chronic bronchitis and community-acquired pneumonia [9], it has also been applied to other local infections by researchers. To date, only one publication by Islinger *et al*. evaluated the free gemifloxacin concentrations in skeletal muscle [10]. However, their study was performed on healthy volunteers rather than infected subjects. In addition, the method the authors employed to obtain the free drug concentrations in plasma according to the plasma protein binding rate may result in inaccurate estimation [11]. Based on this background, we aimed to investigate the muscle distribution of gemifloxacin in infected mammals.

In the present study, *in vivo* MD coupled with LC-MS/MS was established to evaluate the concentrations of unbound gemifloxacin in plasma and skeletal muscle for both MRSA-infected and healthy rats. Thus, the PK profiles of free gemifloxacin in plasma and skeletal muscle can be elucidated, and the tissue penetration ability of gemifloxacin can be clarified.

## Materials and methods

### Chemicals, reagents and bacterial strain

**Fig 1:**
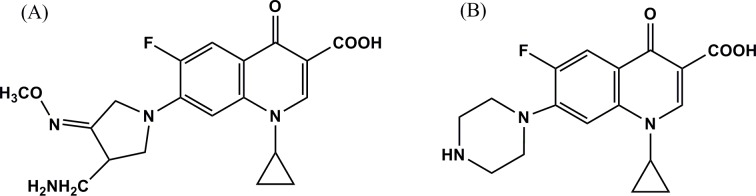
The chemical structures of gemifloxacin (A) and ciprofloxacin (B).

Gemifloxacin mesylate for injection (purity > 99.7%) was supplied by Livzon Pharmaceutical Group Inc. (Zhuhai, China). Ciprofloxacin (purity > 99%), which served as an internal standard (IS), was purchased from the National Institutes for Food and Drug Control (Beijing, China). The chemical structures of gemifloxacin and ciprofloxacin are shown in Fig 1. Pentobarbital sodium (purity > 99%) was obtained from Sigma-Aldrich Corp. (St Louis, MO, USA). HPLC-grade methanol was purchased from Fisher Scientific (Fair Lawn, NJ, USA). Ultrapure water was obtained from a Millipore water purification system (Bedford, MA, USA). Other chemicals were of analytical grade. Ringer’s solution comprising 145 mM NaCl, 4.02 mM KCl, and 2.24 mM CaCl_2_ was prepared freshly. MRSA strain ATCC 43300 from the CAMS Collection Center of Pathogen Microorganisms (CCPM) was used in this study. Mueller-Hinton broth and agar used for bacterial culture and antimicrobial susceptibility tests were purchased from Becton, Dickinson and Company (Franklin Lakes, NJ, USA).

### Preparation of calibration standards and quality controls

A stock solution of gemifloxacin was prepared by dissolving gemifloxacin in Ringer’s solution at a concentration of 1 mg/mL. Calibration standards (2, 4, 8, 16, 32, 64, 128 and 256 ng/mL) and quality control (QC) samples (4, 40 and 200 ng/mL) were prepared by subsequent dilutions of stock solution with blank dialysate. The IS stock was prepared at a concentration of 1 mg/mL and subsequently diluted to 10 ng/mL in methanol as the working solution. Both the stock and working solutions were stored at -20°C. All solutions were kept from light.

### Sample preparation procedure

Dialysate samples were thawed at room temperature before analysis. The mixture of 20 μL sample or standard, 20 μL IS working solution and 160 μL methanol–water solution (1:1, v/v) was vortexed for 1 min, transferred to a clean autosampler insert, and directly injected into the LC-MS/MS system for determination.

### Instrumentation and analytical conditions

**Fig 2:**
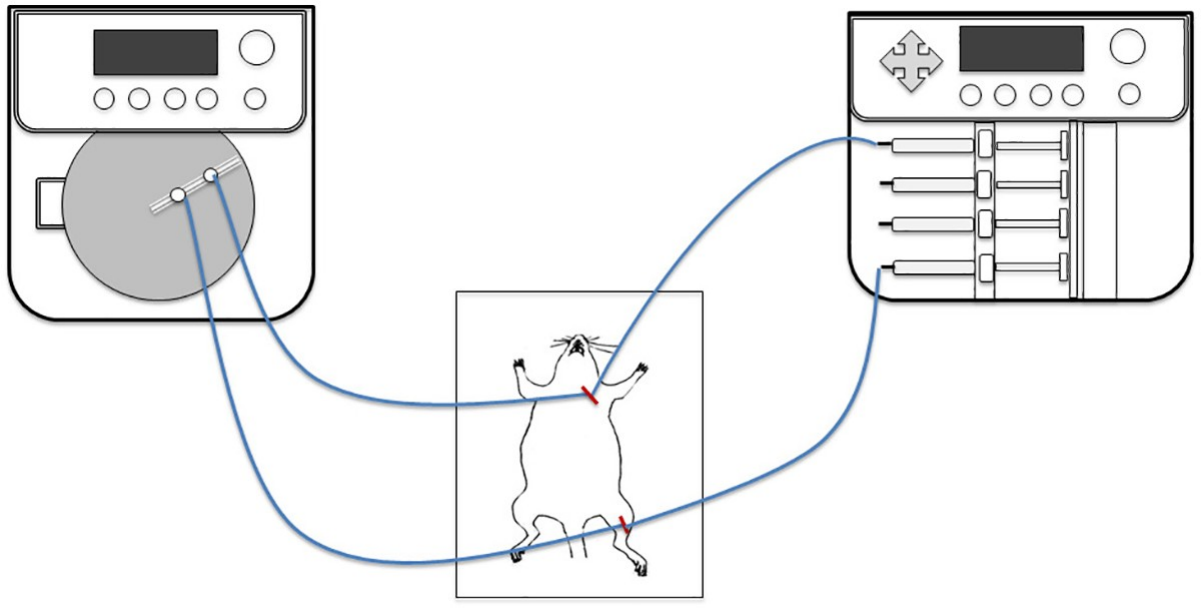
The configuration of the microdialysis system.

To collect free gemifloxacin in the interstitial space fluid at the target tissue [12–13], commercially available MD probes (membrane length, 10 mm; membrane diameter, 0.5 mm; membrane materials, polyarylethersulfone; molecular cutoff, 20 kDa; CMA 20, CMA/Microdialysis AB, Stockholm, Sweden) were employed. The MD system was connected to a microinfusion CMA400 syringe pump (flow rate range, 1 nL/min to 1 mL/min; flow rate accuracy, ±1%; flow rate reproducibility, ±0.1%; CMA/Microdialysis AB, Stockholm, Sweden) and a CMA470 refrigerated fraction collector. Each probe was coupled to a gas-tight microliter syringe (500 μl; Hamilton Company, Reno, NV, USA) to provide the perfusion solution. The configuration of the MD system is presented in Fig 2.

LC-MS/MS was performed using a high-performance liquid chromatography (HPLC) system (Shimadzu, Kyoto, Japan), consisting of an LC-20Adxr pump, a SIL-20Acxr autosampler and a CTO-20AC column oven, coupled to an AB SCIEX Qtrap 6500 mass spectrometer (Foster, CA, USA) with an electronic spray ion (ESI) source. Mass spectrometric analysis was acquired in positive ion mode.

Chromatographic separation was achieved using an XTerra MS C18 column (i.d. 50 mm×2.1 mm, 3.5 μm; Waters, Wexford, Ireland) at 35°C. Mobile phase A and B consisted of water and methanol, respectively, with both containing 0.2% formic acid. The gradient elution was programmed as follows: 10% B maintained for 1 min, linearly increased to 70% in 1.5 min and maintained for 1 min, decreased to 10% in 0.1 min and equilibrated to the end of analysis. The total run time was 4.5 min at a flow rate of 0.4 mL/min. Analysis was performed by monitoring the transitions of (m/z)^+^ 390.1→313.0 (collision energy 40 eV) and (m/z)^+^ 332.2→314.1 (collision energy 27 eV) for gemifloxacin and IS, respectively, in multiple reactions monitoring (MRM) mode. The MS/MS parameters were set as follows: ion spray voltage, 4.5 KV; source temperature, 550°C. Data acquisition and processing were performed by Analyst (Version 1.6.2, AB SCIEX Instruments, Foster, CA, USA).

### Method validation

The FDA guideline was followed for the validation of this bioanalytical method [14]. Analytical performance parameters, including selectivity, linearity, lowest limit of quantitation, accuracy, precision, and stability of gemifloxacin under various storage conditions were evaluated.

The selectivity of the method was evaluated by comparing the chromatograms of blank dialysates from six individual rats with the corresponding dialysates spiked with gemifloxacin to investigate the interference from endogenous material.

The calibration equation was obtained from a linear least-squares regression of the peak area ratio of gemifloxacin to IS against the corresponding nominal concentration (ranging from 2 to 256 ng/mL) with a weighting index of 1/x. The lowest concentration in the calibration curve with qualified precision (relative standard deviation (R.S.D.) % < 20%) and accuracy (80–120%) was defined as the lower limit of quantitation (LLOQ). The R.S.D. within ±15% for the back calculation from the nominal concentrations was considered acceptable for other levels.

Six replicates of QC standards at three different concentrations (4, 40 and 200 ng/mL) prepared within the same day or on three consecutive days were analyzed for the determination of intra-day and inter-day accuracy and precision. Accuracy was assessed by (mean measured concentration) / (nominal concentration) × 100%, and the precision was expressed in terms of R.S.D.%. The accuracy values should be within 85% to 115%, and the precision values should not exceed 15%.

Six different sources of blank dialysates were used to assess the matrix effect of gemifloxacin at three QC levels. Matrix effects were assessed by comparing the peak area of gemifloxacin added to extracted blank dialysates with that of gemifloxacin added to neat solution. A matrix effect between 80% and 120% is considered ideal. Sample extraction was not involved in the sample preparation process of this study due to the purity of the dialysates; therefore, extraction recovery was not performed in the method validation.

The analysis of six replicates of QC standards at three concentrations maintained at ambient temperature for 12 h, in storage at −80°C for 2 weeks, after three freeze-thaw cycles, and placed in the autosampler after processing at 4°C for 24 h was applied to the stability evaluation of gemifloxacin.

### Relative recoveries of gemifloxacin

To assess the influence of assay methods and drug concentrations on the relative recovery of the probes, both *in vitro* and *in vivo* methods were employed in this study. Two different extraction efficiency (EE) and retro-dialysis (RD) methods described by Tasso *et al*. [15] were applied to the determination of *in vitro* recovery. EE recovery is defined as the ratio of the concentration in the microdialysate (C_out_) and the concentration in the solution surrounding the probe. RD recovery can be calculated by the concentration ratio of drug lost during its passage through the probe (C_in_—C_out_) and drug entering the probe (C_in_). For *in vivo* evaluation, only the RD method was employed. All methods were carried out at 22 ± 2°C.

#### Assessment of *in vitro* microdialysis

To assess the recovery dependency on drug concentration and dialysis method, three different concentrations (100, 2000 and 20000 ng/mL) were employed to evaluate the *in vitro* recoveries of gemifloxacin according to the EE and RD method.

Blank Ringer’s solution was perfused at a flow rate of 2.5 μL/min continuously through the probes (n = 3), which were inserted into different concentrations of gemifloxacin in Ringer’s solutions. Following 1 h of equilibration, dialysate samples were collected every 30 min. The concentrations of gemifloxacin in the solutions and dialysates were measured by the validated LC–MS/MS method to calculate the recovery by the EE method using the following equation:

$$EE\;(\%)=C_{out}/C_{sol}\times 100$$ (1)

EE (%) is the relative recovery, C_out_ is the drug concentration of the dialysate, and C_sol_ is the concentration of the solution where the probe was placed in.

In contrast to the EE method described above, the probes (n = 3) placed in the tubes containing blank Ringer’s solution were perfused by gemifloxacin solutions at a constant flow rate of 2.5 μL/min to evaluate the recovery by the RD method. The recoveries were calculated as follows:

$$RD\;(\%)=\left(C_{in}-C_{out}\right)/C_{in}\times 100$$ (2)

RD (%) is the relative recovery, C_in_ is the concentration of the perfusate, and C_out_ is the drug concentration of the dialysate.

#### Assessment of *in vivo* microdialysis

To evaluate the probe recoveries in rat skeletal muscle and plasma, probes were inserted through the skeletal muscle and blood vessels [16–17]. To implant the probe in the muscle, an incision was made in the left hind leg skin of an anesthetized rat. Then, a guide cannula was inserted through the skeletal muscle, and the probe was placed inside the guide cannula, which was removed while keeping the probe in place. For the probe implantation in blood, the jugular vein was exposed, an incision was made in the blood vessel, and an MD probe was inserted. The probe was held in place with a tie around the probe shaft and skin.

Gemifloxacin in Ringer’s solutions at different concentrations (100, 2000 and 20000 ng/mL) was pumped through the probes (n = 3) continuously at a flow rate of 2.5 μL/min. After 1 h of equilibration, samples were collected every 30 min for each concentration. The relative loss of gemifloxacin from the perfusate into the extracellular fluid was used to calculate the *in vivo* recovery by Eq (2).

### Plasma protein binding studies

Gemifloxacin plasma protein binding was assessed using Amicon Ultra centrifugal filters (Millipore Corporation; Billerica, MA, USA) with a 30-kDa molecular cutoff. Fortified plasma samples (C_plasma_) at concentrations of 10, 1, and 0.05 μg/mL were incubated at 37 ± 1°C for 30 min, transferred into ultrafiltration tubes, and centrifuged at 2,000 *g* for 40 min to generate ultrafiltrate samples (C_ultrafiltrate_). The protein binding rate (%) was calculated as 100 - (100 × C_ultrafiltrate_/C_plasma_).

### Animal studies

The potential of gemifloxacin to penetrate skeletal muscle was investigated in rats. All animal experiments were approved by the Ethics Committee of the Institute of Medicinal Biotechnology, Chinese Academy of Medical Sciences and Peking Union Medical College, China. Male Sprague-Dawley rats (200 ± 10 g) were purchased from the Academy of Military Medical Sciences (Beijing, China). Rats were housed under environmentally controlled conditions with a room temperature of 22 ± 2°C, humidity of 55 ± 6%, and 12-h light/12-h dark cycle. Rats received free access to food and water. All rats were euthanized with CO_2_ after experiments.

#### Animal model of thigh infection

Rats were rendered neutropenic by injecting cyclophosphamide intraperitoneally on day 1 to day 3 at the dose of 35 mg/kg, and the infection was performed on day 4. Twenty microliters of orbital blood was acquired from day 1 until sacrifice, and leukocytes were counted by an inverted microscope AE31 (Motic China Group Co., Ltd., Xiamen, China). The thigh infection was induced by intramuscular injection of 0.1 mL of *S*. *aureus* ATCC 43300 (approximately 10^7^ colony forming units (CFU)/mL) into the left thigh of rats (n = 12). Gemifloxacin (0.2 mL) at a dose of 18 mg/kg or 0.85% NaCl solution was injected into the lateral tail vein 2 h after thigh infection. Rats in both groups were sacrificed 2 h and 26 h after bacterial inoculation, and the thigh muscle was collected, homogenized in 5 mL sterilized saline, and plated to determine the bacterial load. Bacterial counts were recorded as the log_10_ CFU/thigh [18–19].

#### Pharmacokinetic study

After 3 days of acclimatization, rats were randomly divided into two groups (MRSA-infected group, n = 6; healthy control group, n = 6). Animals were anesthetized with pentobarbital sodium (60 mg/kg, i.p.) and immobilized on a dissecting board in the supine position. Anesthesia was confirmed by the absence of reflexes in response to footpad pinching.

MD probes were implanted into plasma and skeletal muscle for sampling as described above. The probes were perfused with Ringer’s solution at a flow rate of 2.5 μL/min from 1 h before drug administration. A single intravenous dose of 18 mg/kg was administered by the lateral tail vein. The dosage was equivalent to 200 mg i.v. or 320 mg oral (dosage of commercial gemifloxacin tablet) dosing in humans based on the body surface area conversion.

MD samples were collected up to 9 h with the sampling program as follows: every 10 min for the first 1 h; 20 min intervals for the following 3 h; and 30 min intervals for the last 5 h with sampling amounts of 25 μL, 50 μL and 75 μL, respectively. Dialysate samples were stored at −80°C until analysis. Twenty microliters of dialysates was briefly mixed with 20 μL IS working solution and 160 μL methanol–water solution (1:1, v/v), vortexed for 1 min, transferred to a clean autosampler insert, and directly injected into the LC-MS/MS system for determination.

#### Tissue uptake study

Sixteen MRSA-infected rats were randomly divided into four groups of four animals each, corresponding to 0.167, 2, 4, and 9 h postdose for muscle collection. Gemifloxacin was administered to rats intravenously at a dose of 18 mg/kg. Rats were sacrificed at the sampling time, and muscle samples were removed, rinsed with normal saline solution (0.9%, w/v), wiped dry, and weighed immediately. The tissues were kept frozen at −80°C until analysis.

### Data analysis

PK parameters were assessed by Phoenix v. 64 (Pharsight Corp., Mountain View, CA, USA) employing a non-compartmental model. The differences in MD probe recoveries among three concentrations and two methods (RD and EE) were evaluated by one-way ANOVA employing SPSS 16.0. Student’s *t*-test was conducted on gemifloxacin exposures between healthy rats and MRSA-infected rats. Paired samples *t*-test was conducted on the PK parameters between muscle and plasma for statistical comparison. A *P*-value < 0.05 was considered statistically significant.

## Results

### Method validation of LC-MS/MS analysis

**Fig 3:**
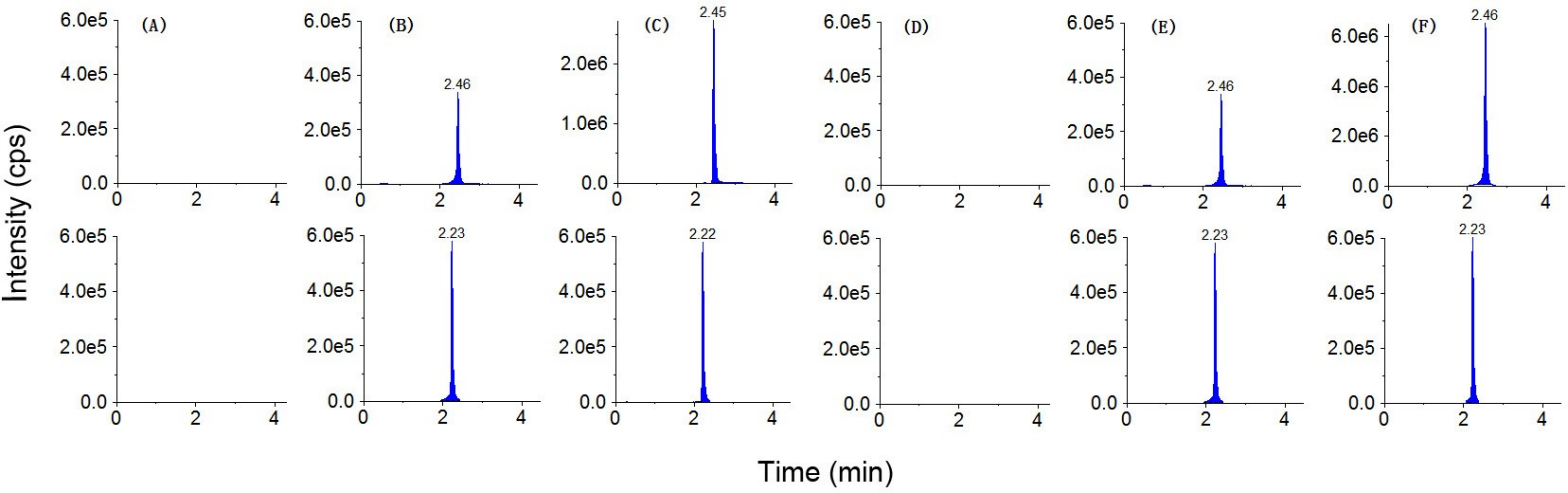
LC–MS/MS chromatograms of gemifloxacin (upper row) and ciprofloxacin (bottom row). (A) Blank dialysate of skeletal muscle; (B) blank skeletal muscle dialysate spiked with gemifloxacin (40 ng/mL); (C) dialysate of skeletal muscle collected 4 h after the administration of gemifloxacin, i.v. (18 mg/kg); (D) blank dialysate of plasma; (E) blank plasma dialysate spiked with gemifloxacin (40 ng/mL); (F) dialysate of plasma collected 4 h postdose.

Selectivity was evaluated by comparing the chromatograms of blank dialysate, blank dialysate spiked with gemifloxacin (40 ng/mL), and dialysate of skeletal muscle or plasma collected 4 h postdose. As shown in Fig 3, no significant endogenous interference was displayed at the retention time of gemifloxacin or ciprofloxacin (IS) in MRM scan mode.

The standard calibration curves showed good linearity with a typical equation of *y* = 0.0307*x* - 0.049 (*R*^2^ = 0.9982, Weight: 1/*x*) over the range of 2−256 ng/mL. All analytical results exhibited less than 15% deviation from the nominal concentrations. The LLOQ was 2 ng/mL with acceptable precision and accuracy.

The results of intra-day and inter-day precision and accuracy are presented in Table 1. R.S.D. (%) values were no more than 14.15% and 11.33% for intra-day and inter-day precision, respectively. The accuracy ranged from 88.25% to 107.73% for all QC levels. The accuracies and precisions for the tested levels were all within the defined acceptable criteria.

**Table 1:** Accuracies and precisions of gemifloxacin QC samples.

Nominal concentration (ng/mL)		Intra-day		Inter-day	
		Accuracy (%,n = 6)	RSD (%,n = 6)	Accuracy (%, n = 18)	RSD (%, n = 18)
Day 1	4	107.73	7.75	105.49	6.38
Day 2		103.32	7.72		
Day 3		105.43	3.69		
Day 1	40	88.25	4.43	91.22	8.58
Day 2		93.28	14.15		
Day 3		92.14	7.18		
Day 1	200	95.14	11.88	94.65	11.33
Day 2		98.98	12.68		
Day 3		89.84	9.45		

As shown in Table 2, the matrix effects were between 91.32% and 102.16%, which suggests the absence of ion suppression or ion enhancement.

**Table 2:** The matrix effect quantitative values of gemifloxacin in dialysates (n = 6).

Nominal concentration (ng/mL)	Matrix effect (%)	
	Mean (%)	RSD (%)
4	102.16	7.56
40	91.32	8.39
200	99.36	7.45

The results of the stability study summarized in Table 3 showed that gemifloxacin did not degrade after being placed at room temperature for 12 h or stored at −80°C for 2 weeks in dialysate samples. Gemifloxacin was also stable in autosampler at 4°C for 24 h and after undergoing three freeze-thaw cycles.

**Table 3:** Stability data for gemifloxacin (n = 6).

Nominal concentration (ng/mL)	%Theoretical			
	12 h ambient	2 week frozen (−80°C)	3 freeze / thaw cycle	24 h in autosampler (4°C)
4	94.48±8.69	102.16±7.56	97.87±4.32	96.40±1.75
40	100.17±1.53	91.32±8.39	101.45±7.38	92.42±3.41
200	97.40±4.04	99.36±7.45	91.67±3.32	95.98±5.25

### *In vitro* and *in vivo* recoveries of microdialysis

The influence of the gemifloxacin concentration and method employed on probe recovery was evaluated. The relative *in vitro* recoveries were 22.15% ± 1.10%, 24.63% ± 2.28% and 23.27% ± 2.01% in the EE method for gemifloxacin concentrations of 100, 2000 and 20000 ng/mL, respectively. The corresponding recoveries measured by the RD method were 23.56% ± 2.77%, 24.28% ± 2.32% and 25.80% ± 2.30%, respectively. There was no significant difference (*P*>0.05) among the three different drug concentrations or two dialysis methods.

The average *in vivo* recoveries measured by the RD method were 23.21% ± 3.42% for skeletal muscle and 20.62% ± 3.19% for plasma. The *in vivo* recovery values above were employed to back calculate the real gemifloxacin concentrations in rat plasma and skeletal muscle.

### Plasma protein binding

The *in vitro* protein binding rates of gemifloxacin over three different plasma concentrations were tested. The mean fraction bound to plasma proteins was 74.5% ± 5.7% with no concentration dependence.

### Thigh infection model

**Fig 4:**
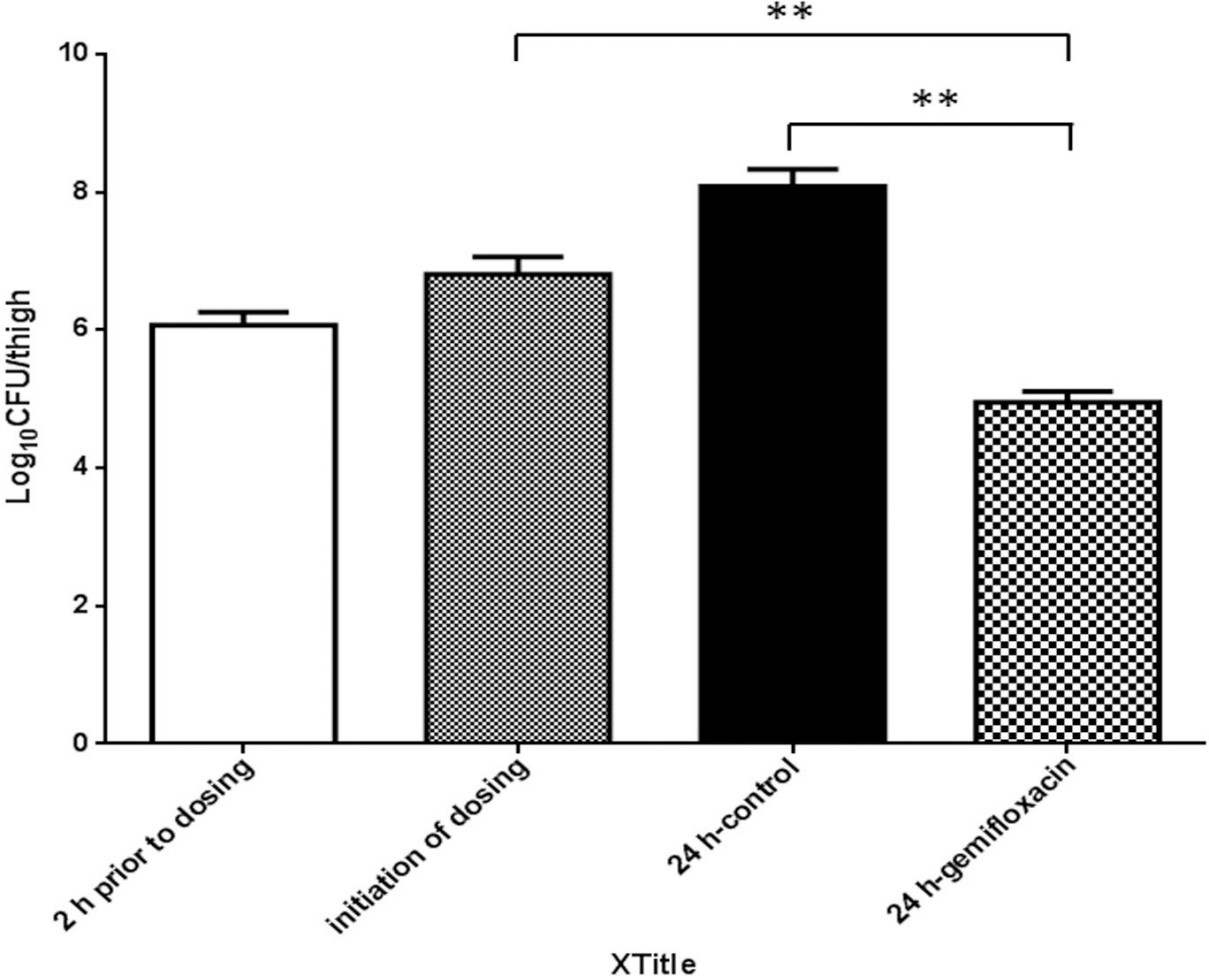
The bacterial density in the thigh infection model (Mean*±* S.D., n = 4).

The thigh infection model was successfully established in neutropenic rats. As presented in Fig 4, the bacterial density was 6.07±0.19 log_10_ CFU/thigh. The bacterial density taken from control animals prior to initiation of dosing was 6.80±0.26 log_10_ CFU/thigh, which increased to 8.08±0.25 log_10_ CFU/thigh after 24 h in untreated controls. A single dose of gemifloxacin at 18 mg/kg produced an approximate 2 log_10_ CFU/thigh reduction at 24 h after treatment compared with that before treatment.

### Pharmacokinetic and tissue penetration study of gemifloxacin

To investigate the penetration ability of gemifloxacin into skeletal muscle, free gemifloxacin concentration was determined in skeletal muscle and plasma by MD combined with LC-MS/MS after an 18 mg/kg i.v. bolus dose.

**Fig 5:**
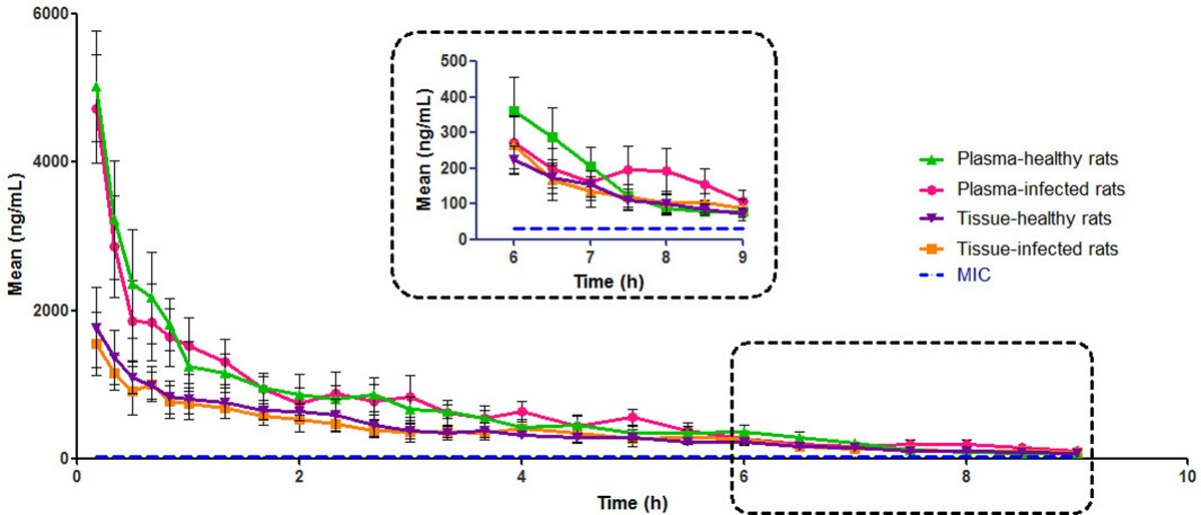
Free gemifloxacin concentrations in plasma and skeletal muscle after i.v. administration of 18 mg/kg to infected rats (Mean*±* S.D., n = 6) and healthy rats (Mean*±* S.D., n = 6). The final phase scale is expanded in the inset.

The profiles of free gemifloxacin from 0 to 9 h in plasma and skeletal muscle in both infected and healthy rats are shown in Fig 5. The MIC of gemifloxacin for ATCC 43300 conducted by broth microdilution in cation-adjusted Muller-Hinton broth according to the Clinical and Laboratory Standards Institute (CLSI) protocol [20] was 0.03 mg/L. Free gemifloxacin concentrations were above the MIC during the 9-h sampling period in plasma and muscle as shown in Fig 5. The concentrations of last sampling point (C_9h,plasma_ and C_9h, muscle_) were more than 2 folds of MIC. The rapid distribution of gemifloxacin from plasma to muscle and markedly lower free levels of gemifloxacin in skeletal muscle than in plasma were observed. The main PK parameters are summarized and presented in Table 4.

**Table 4:** Pharmacokinetic parameters^a^ of gemifloxacin in plasma and skeletal muscle following an intravenous dose of 18 mg/kg (n = 6).

Compartment	AUC_0-9_ (h*ng/mL)	AUC_0-∞_ (h*ng/mL)	T_1/2_ (h)	T_max_ (h)	C_max_ (ng/mL)	Vd (mL/kg)	CL (mL/h/kg)
Plasma (infected rats)	7068.32± 1964.19	7542.42± 2153.95	2.12± 0.24	0.17± 0.00	4726.20± 729.82	7964.85± 3127.27	2560.83± 775.17
Muscle (infected rats)	3641.50± 915.65	3920.44± 1010.14	2.10± 0.50	0.37± 0.30	1675.19± 584.57	ND ^b^	ND ^b^
Plasma (healthy rats)	6927.49± 1714.86	7076.15± 1674.23	1.33± 0.43	0.17± 0.00	5027.43± 746.97	5234.53± 2472.32	2630± 547.64
Muscle (healthy rats)	3774.72± 700.36	3962.03± 752.62	1.87± 0.46	0.17± 0.00	1766.37± 541.64	ND ^b^	ND ^b^

^a^Abbreviations: AUC_0–9_, area under curve from time zero to 9h; AUC_0-∞_, area under curve from time zero to infinity; T_1/2_, the plasma elimination half-life; T_max_, the time to maximum concentration; C_max_, the maximum concentration; Vd, volume of distribution; CL, Clearance.

^b^ ND, not determined.

The corresponding PK parameters for plasma calculated using the noncompartmental model were as follows: T_1/2_ = 2.12 ± 0.24 h, AUC_0–9_ = 7068.32 ± 1964.19 ng h/mL, AUC_0–∞_ = 7542.42 ± 2153.95 ng h/mL and C_max_ = 4726.20 ± 729.82 ng/mL in MRSA-infected rats; and T_1/2_ = 1.33 ± 0.43 h, AUC_0–9_ = 6927.49 ± 1714.86 ng h/mL, AUC_0–∞_ = 7076.15 ± 1674.23 ng h/mL and C_max_ = 5027.43 ± 746.97 ng/mL in healthy rats. The above parameters were in accordance with the reported data taking into account the protein binding rate [21–22].

Compared with the results for plasma, the results for skeletal muscle revealed a significantly lower AUC_0–9_ and C_max_ (*P* < 0.05) in both healthy (AUC_0–9_ = 3774.72 ± 700.36 ng h/mL and C_max_ = 1766.37 ± 541.64 ng/mL) and infected rats (AUC_0-9_ = 3641.50 ± 915.65 ng h/mL and C_max_ = 1675.19 ± 584.57 ng/mL). The tissue penetration factor (expressed as the mean ratio of the AUC_0-9_ for muscle to the AUC_0-9_ for plasma) for infected rats and healthy rats was 0.52 and 0.54, respectively.

The difference in tissue exposure of gemifloxacin between healthy and MRSA-infected rats was also investigated and analyzed. No significant difference was observed (*P*>0.05) between healthy and MRSA-infected rats in plasma or thigh muscle.

### Tissue distribution

The tissue distribution study was performed on infected rats. The concentrations of gemifloxacin in homogenized muscle (C_m_) are presented in Table 5. To elucidate the distribution of gemifloxacin between the interstitial space and tissue cells, the intracellular concentration (C_intra_) and the concentration in the muscle interstitial fluid (C_isf_, total amount of unbound and bound concentrations) were calculated by the following equations reported by Araki and shown in Table 5 [23].

$$C_{intra}=\left(C_{m}-C_{isf}\times 0.119\right)/0.881$$ (3)

$$C_{isf}=C_{isf,u}+C_{isf,b}=C_{isf,u}+C_{p,b}\times 0.6$$ (4)

**Table 5:** Muscle distribution parameters of gemifloxacin in MRSA-infected rats following an intravenous dose of 18 mg/kg (n = 4).

Sampling time	C_m_ (ng/mL)	C_isf,u_ (ng/mL)	C_p,u_ (ng/mL)	Unbound fraction	C_p,b_ (ng/mL)	C_isf,b_ (ng/mL)	C_isf_ (ng/mL)	C_intra_ (ng/mL)
10min	22825.0± 943.0	1546.5± 427.1	4726.2± 729.8	0.255	13807.9	8284.8	9831.3	24580.1
2h	8552.5± 732.3.0	529.2± 164.0	749.9± 213.2		2190.7	1314.4	1843.6	9458.7
4h	2725.0± 345.9	416.0± 108.7	640.0± 132.9		1869.9	1121.9	1537.9	2885.3
9h	346.2± 60.7	86.6± 25.5	107.9± 31.1		315.2	189.1	275.7	355.8

C_isf,u_ is the unbound concentration in interstitial fluid obtained by MD, C_isf,b_ is the bound concentration in interstitial fluid, and C_p,b_ is the bound concentration in plasma.

The constants of 0.119 and 0.881 were the reported ratios for the interstitial and intracellular volumes, respectively, in rat muscle. A constant of 0.6 was the reported albumin ratio of interstitial fluid to plasma.

As shown in Table 5, the C_m_ obtained using the homogenized tissue, at a comparable level of C_intra,_ was higher than C_isf_. The result indicated that gemifloxacin is mainly distributed in tissue cells rather than the interstitial space.

## Discussion

Most PK studies on antimicrobial agents are based on the measurement of total plasma concentration. However, antibacterial activity can be exerted only by the drugs of the unbound fraction in the interstitial fluid where the infections occur. In local infection, PK profiles for plasma and target tissues may be markedly different. Therefore, the prediction of antimicrobial efficacy based on plasma PK data may result in overestimation of the dose regimen and failure of antibiotic therapy. Regulatory authorities such as the FDA and European Agency for the Evaluation of Medicinal Products advocate that the concentrations of antibacterial at the target site should be determined.

Islinger *et al*. found that free gemifloxacin concentrations in muscle were persistently higher than those in plasma over the 10-h study period in healthy volunteers with a penetration factor of 1.7, which was different from the PK characteristics of other fluoroquinolones [24–25].

In the present study, MD combined with LC-MS/MS was applied to determine free gemifloxacin in plasma and skeletal muscle simultaneously in rats. MD is a minimally invasive technique to achieve unbound drug sampling in the tissue of interest and was applied to the tissue distribution investigation in this study. Because of the high purity of the dialysate, sample extraction and the corresponding validation were not required for MD samples. Instead of extraction recovery, the relative recoveries of MD probes were evaluated. There was no significant difference among the three different drug concentrations, which showed that probe recovery was independent of the concentrations investigated. No significant difference in recoveries was found between the EE and RD methods, indicating that the RD method can be employed for the assessment of *in vivo* recoveries. Therefore, the *in vivo* recovery values obtained by the RD method were employed to back calculate the real gemifloxacin concentrations in rat plasma and skeletal muscle.

The concentration-time curve in our study indicated that free gemifloxacin concentrations were above MIC during the 9-h sampling period in both plasma and muscle, which is promising for the treatment of MRSA infections.

A key finding of our study is the significantly lower exposure of gemifloxacin in skeletal muscle than in plasma for both healthy and MRSA-infected rats with penetration factors less than 0.6. This finding represented a completely different profile than the results reported by Islinger *et al*. [10]. The infection status may cause corresponding pathological and physiological changes and result in altered tissue penetration [26]. Moreover, membrane transport processes such as influx and efflux play a key role in the tissue distribution of antibacterials, and the expression and activity of P-glycoprotein, a membrane transporter, can be altered by the influence of infection and inflammation [27]. Therefore, we speculated that the tissue uptake of gemifloxacin, as a P-glycoprotein substrate [28], may also be affected by the infection status. To investigate the influence of infection status on the tissue penetration of gemifloxacin, both healthy and infected rats were included in the experimental design. However, our results proved that there was no significant difference in gemifloxacin exposure between healthy and MRSA-infected rats in plasma or thigh muscle. Our findings also proved that infected rats can be replaced by healthy rats in the PK/PD studies of gemifloxacin in rats, which will be highly convenient for *in vivo* PK/PD studies.

Several other reasons can account for this difference. First, the species difference between rats and humans may lead to different tissue distributions of gemifloxacin. This finding also suggested that the extrapolation of PK/PD data from rats to humans should be approached with caution. The sampling method may be another factor that affected the final results. Different methods were employed for the determination of free gemifloxacin in plasma in our study and Islinger’s study. The free concentrations in plasma calculated based on the total drug concentration determined by protein precipitation extraction and the plasma protein binding rate may be less accurate than those achieved directly by MD.

Similar to other fluoroquinolones, gemifloxacin is a concentration-dependent antibacterial, and the ratio of AUC_0-24_ to the MIC_90_ is the most relevant PK/PD parameter to clinical efficacy [29–30]. The relatively low AUC of gemifloxacin in skeletal muscle compared with that in plasma indicated that the dosing regimen according to drug levels in plasma for the treatment of local tissue infections is unreasonable and might result in insufficient dosage or even drug resistance. Therefore, it is important to precisely determine the free drug levels in target tissues to confirm the antimicrobial efficacy.

The present study also measured and compared the concentrations of gemifloxacin from homogenized tissue and interstitial fluid in infected rats. Tissue homogenization is the traditional method for tissue distribution studies. However, bacterial infections are usually restricted to extravascular interstitial spaces, and only the unbound fraction exhibits antimicrobial effects. Therefore, it is important to evaluate the distribution in tissue interstitial space and intracellular space separately, especially the unbound concentrations in tissue interstitial fluid, for an antimicrobial agent. The result of C_intra_≈C_m_>C_isf_ for gemifloxacin, consistent with the previously reported result of ciprofloxacin [23], further demonstrated that compared with the muscle concentration from tissue homogenization, the unbound concentration of gemifloxacin in tissue interstitial fluid measured using MD is extremely valuable for the evaluation of antimicrobial effects.

The present work is the first to investigate the penetration ability of free gemifloxacin in infected skeletal muscle in rats. A rodent model for the PK/PD study of gemifloxacin in local infection was also established.

## Conclusions

In conclusion, this validated method was successfully applied to quantify free gemifloxacin concentrations in rat plasma and thigh muscle and provides the possibility to investigate the real concentrations of gemifloxacin in different parts of the body simultaneously, which would shed light on the dosing regimen optimization of gemifloxacin. The significantly lower free gemifloxacin concentrations in skeletal muscle than in plasma in MRSA-infected rats suggested that the dosing regimen according to drug levels in plasma to the treatment of local tissue infection is unreasonable.

## Abbreviations

AUC: area under the concentration-time curve
CL: Clearance
C_max_: the maximum concentration
EE: extraction efficiency
IS: internal standard
i.v.: intravenous
LLOQ: lower limit of quantitation
MD: microdialysis
MIC: minimum inhibitory concentration
MRSA: methicillin-resistant *Staphylococcus aureus*
PK: pharmacokinetics
PK/PD: pharmacokinetic/pharmacodynamic
RD: retro-dialysis
T_1/2_: half-life
T_max_: the time to maximum concentration
Vd: volume of distribution
